# Is the drug-induced hypersensitivity syndrome (DIHS) due to human herpesvirus 6 infection or to allergy-mediated viral reactivation? Report of a case and literature review

**DOI:** 10.1186/1471-2334-10-49

**Published:** 2010-03-06

**Authors:** Ivan Gentile, Maria Talamo, Guglielmo Borgia

**Affiliations:** 1 Department of Infectious Diseases and Legal Medicine-Section of Infectious Diseases (Ed 18) - University of Naples "Federico II", via S Pansini, 5 I-80131 Naples Italy

## Abstract

**Background:**

Drug-Induced Hypersensitivity Syndrome (DIHS) is a severe and rare systemic reaction triggered by a drug (usually an antiepileptic drug).  We present a case of DISH and we review studies on the clinical features and treatment of DIHS, and on its pathogenesis in which two elements (Herpesvirus infection and the drug) interact with the immune system to trigger such a syndrome that can lead to death in about 20% of cases.

**Case presentation:**

We report the case of a 26-year old woman with fever, systemic maculopapular rash, lymphadenopathy, hepatitis and eosinophilic leukocytosis. She had been treated with antibiotics that gave no benefit. She was taking escitalopram and lamotrigine for a bipolar disease 30 days before fever onset. Because the patient's general condition deteriorated, betamethasone and acyclovir were started. This treatment resulted in a mild improvement of symptoms. Steroids were rapidly tapered and this was followed with a relapse of fever and a worsening of laboratory parameters. Human herpesvirus 6 (HHV-6) DNA was positive as shown by PCR. Drug-Induced Hypersensitivity Syndrome (DIHS) was diagnosed. Symptoms regressed on prednisone (at a dose of 50 mg/die) that was tapered very slowly. The patient recovered completely.

**Conclusions:**

The search for rare causes of fever led to complete resolution of a very difficult case. As DIHS is a rare disease the most relevant issue is to suspect and include it in differential diagnosis of fevers of unknown origin. Once diagnosed, the therapy is easy (steroidal administration) and often successful. However our case strongly confirms that attention should be paid on the steroidal tapering that should be very slow to avoid a relapse.

## Background

Drug-Induced Hypersensitivity Syndrome (DIHS) is a life-threatening systemic reaction characterized by rash, fever, hepatitis, lymphadenopathy and leukocytosis with eosinophilia. It is triggered by a drug (usually an antiepileptic drug) started 3 weeks-3 months before symptoms onset [1]. Several drugs have been associated with DIHS, namely, carbamazepine, phenytoin, phenobarbital, lamotrigine, zonisamide, allopurinole, dapsone, minocycline, salazosulphapyridine. Abacavir and nevirapine have been associated with a DIHS caracterized by some peculiar symptoms [1].

It is a rare event: the incidence ranges from 1.2 to 6 per million person-years. Between 1:1000 to 1:10000 patients treated with phenytoin develop DIHS.

It is even rarer in lamotrigine-treated patients in the setting of mood disorders [2]. Adults are more likely to be affected than children, while there is no sex predilection [1].

Treatment with corticosteroids is often successful. However DIHS leads to death in about 20% of cases.

We report the case of a 26-year old woman with fever, maculopapular rash, lymphadenopathy, hepatitis and eosinophilic leukocytosis. She had started an antiepileptic drug (lamotrigine) for a bipolar disease 30 days before fever onset. Moreover we review studies on diagnosis and treatment of DIHS, and on its pathogenesis in which two elements (herpesvirus infection and the drug) interact with the immune system to trigger the syndrome.

## Case presentation

A 26-year-old woman was admitted to hospital because of fever. She was diagnosed with von Willembrandt disease in childhood and had undergone additive mastoplasty in 2000. She was diagnosed with bipolar disease when she was 16 years old and had been taking escitalopram and lamotrigine 30 days before fever onset. This treatment was stopped after 4 days of fever. Twelve days before hospital admission she presented fever (maximum: 39.5°C), asthenia, nausea, myalgia and arthralgia, and was treated with cefixime 400 mg bid. After the third dose, a systemic and itchy rash appeared. She was then treated with betametason 1 mg and fenoxifenadine 120 mg for one day, 11 days before hospital admission. Fever and other symptoms persisted despite antibiotic and corticosteroid treatment. She was then treated with azithromycin 500 mg qd for 3 days.

Upon hospital admission, she complained of the above-indicated symptoms. Medical examination showed a maculopapular rash on the face, neck, trunk, and superior and inferior limbs; hard, tender lymph nodes measuring 2-3 cm in diameter bilaterally at retronucal, laterocervical, inguinal and axillary sites; hepatomegaly. Laboratory results showed eosinophilic leukocytosis (31,770/mmc; 18% eosinophils); increase of both CD4 and CD8 T lymphocytes (CD4 = 3,674/mmc and CD8 = 5,759/mmc); high aminotransferase levels (alanine aminotransferase [ALT] and aspartate aminotransferase [AST] were 19-fold and 14-fold normal values, respectively), high bilirubin level (2.84 mg/dL); and low prothrombin activity (39%). Markers for Hepatitis A virus, Hepatitis B virus, Hepatitis C virus were negative. Immunoglobulin-M against herpesvirus I and II, Epstein Barr virus (EBV), Cytomegalovirus (CMV), Rubella, Adenovirus, Coxsackievirus, Influenza virus A/B, parainfluenza virus, antibodies against Borrelia burgdoferi, Rickettsia conori, Rickettsia typhi, Chlamydia trachomatis, Leishmania infantum were negative as was Vidal-Wright serodiagnosis. Culture of blood, pharynx, urine, feces, and parasitological examination of feces showed no infection. Antinuclear antibodies and thyroid hormones were normal. Pathological examination of right axillary lymph nodes showed no malignancy. The numbers of mature T cells and dendritic cells (positive for S-100 protein and CD 68) were increased; conversely B-cell-dependent areas (positive for CD 20) were scarce and confined to the cortex, and there were some eosinophils. Notably, there were foam and hydropic cells, as is seen in lymphadenopathy induced by such inertial material as silicon. Bone marrow examination was unremarkable, and culture of bone marrow blood was negative. A computerized scan showed hepatomegaly and no other pathological sign.

At day 2 post admission in hospital, because the patient's general condition deteriorated, betamethasone 8 mg i.v. and acyclovir 250 mg i.v. every 8 hours for 8 days were started as empirical treatment together with infusions of fresh frozen plasma, physiological solutions, and proton pomp inhibitors. This treatment resulted in a mild improvement of symptoms. After 5 days of treatment, we tapered steroids from 8 mg to 3 mg i.v. in one week. This was associated with a relapse of fever and an increase of aminotransferase levels and eosinophils.

Differential diagnosis involved lymphoproliferative diseases, autoimmune diseases/vasculitis, infectious diseases and allergic reactions to drug.

At day 11 post admission in hospital, we performed anti-human herpesvirus 6 IgG and IgM that tested positive and Human herpesvirus 6 (HHV-6) DNA (real time PCR) in blood that tested positive (8590 cp/mL). We suspected a single disease that included all these diseases, namely Drug-Induced Hypersensitivity Syndrome (DIHS), also called drug reaction with eosinophilia and systemic symptoms (DRESS) syndrome, anticonvulsant hypersensitivity syndrome (AHS), or drug induced pseudolymphoma. Based on diagnostic criteria (see below), we made a diagnosis of DIHS and prescribed prednisone 50 mg/die. Fourteen days after admission to hospital, the fever had regressed and there was an improvement of clinical and laboratory parameters. Steroidal tapering was very slow (5% each 2 weeks). HHV-6 viremia became negative after 50 days of prednisone treatment. At present, 18 months after hospitalization, all parameters are normal and the patient's general condition is good.

### Pathogenesis

Necessary elements for DIHS are: (1) the drug; (2) the virus; and (3) their interplay with the immune system. The drugs associated with DIHS include several anti-epileptic drugs (see above). However, cross-sensitivity between antiepileptic and tricyclic antidepressant agents [3] or among different aromatic anticonvulsants [4] have been reported in the setting of DIHS. A genetic predisposition has been correlated to DIHS. Aromatic anticonvulsants (phenytoin, phenobarbital and carbamazepine) are metabolized partially by the cytochrome P-450 system to reactive aromatic epoxide. Patients with DIHS have a defect of this detoxification system, which suggests that a reactive metabolite or a high level of the drugs play a role in triggering the immune response [5,6]. Relatives of DIHS patients have this detoxification defect, and may therefore be at an increased risk for DIHS. The abnormal detoxification of phenytoin is thought to be inherited in autosomic co-dominant fashion [7].

DIHS has been associated with HHV-6 infection [8]. The syndrome has also been associated with reactivation of other members of the herpesvirus family [9], Human herpesvirus 7 (HHV-7) [8], EBV [10] and CMV [11]. Herpesviruses can reactivate in DIHS in a sequential order as occurs in graft-versus-host disease [12,13].

How is HHV-6 acquired? HHV-6 infects nearly all humans by age 2 years [14]. Most infections arise through the exchange of infected saliva during the first years of life, although perinatal transmission can also occur. It has recently been shown that HHV-6 DNA can be chromosomally integrated into host DNA, and chromosomally integrated HHV-6 is thought to be the major mode of congenital infection [15]. This has been demonstrated also in the setting of DIHS [16].

It is well established that viral infections and drug allergy are the defining features of DIHS; however, which condition is the cause and which is the effect? The trigger could be an allergic reaction to the causative drug that stimulates T cells [17]. T-cell stimulation leads to reactivation of the herpesvirus viral genome harbored in T cells. This would explain the cascade of herpesvirus reactivation in DIHS [12,13]. Alternatively, DIHS could be triggered by herpesvirus reactivation, which is clinically silent. Virus-stimulated T cells can cross-react with the drug thereby leading to expansion of specific T cells. Subsequently, HHV-6 can reactivate heterologous viruses as has been demonstrated in vitro [18]. The temporal relationship between therapy onset and DIHS onset (from 3 weeks to 3 months) suggests that the virus does not play a primary role in the syndrome. Therefore, we favor a primary allergic reaction pathogenesis.

Immunologically, patients with DIHS have decreased total IgG, IgA and IgM levels and B-lymphocytes at disease onset [19,20], whereas there is an expansion of memory T cells that cross-react with both the drug and the virus. It is noteworthy that lymphocyte transformation test, which is used to diagnose drug-specific T-cell responses in the clinical setting, is negative in the first week after DIHS onset and continues to be negative in 90% of patients 2 weeks after disease onset [21]. The test becomes positive 5-7 weeks after disease onset. These results could be due to expansion of regulatory T cells (that suppress proliferation of memory T cells) in the initial stages of the disease and their subsequent decrease via apoptosis [21]. Several cytokines are increased during DIHS. In particular, the levels of TNF-alpha and IL-6, which are typical inflammatory cytokines, are elevated in DIHS before HHV-6 infection [22]. Interestingly, IL-6 levels become undetectable during viral infection and increase again after infection in most patients [22].

### Clinical Features

The first symptom of DIHS is fever, which is often high (38-40°C) and is followed by itchy, patchy erythematous maculae (often with follicular accentuation) that can become confluent. Rash starts on face, upper trunk and upper extremities. In some cases, especially when the causative drug is not discontinued, a severe exfoliative dermatitis occurs. Tender lymphadenopathy (limited to cervical nodes or generalized) is often present. Hepatic involvement is common: hepatomegaly and splenomegaly are often found at clinical examination. Bilateral swelling of the salivary glands with xerostomia has been frequently reported. A typical feature of DIHS is a paradoxical worsening of symptoms after withdrawal of the causative drug [21].

Laboratory data are helpful in diagnosing DIHS: leukocytosis with eosinophilia and atypical lymphocyte count increase are common findings. Both CD4 and CD8 cells are increased in DIHS patients. Similarly, ALT levels are increased in most patients, and hepatitis is often anicteric. Renal involvement is less common. It includes tubulointerstitial nephritis and granulomatous necrotizing angitis [21]. Serum IgG, IgA and IgM levels are often decreased. Another important laboratory feature is the presence of HHV-6 DNA (by PCR) and IgM against HHV-6 in serum.

The death rate of DIHS is 20% [23] and it is associated with older age, renal involvement, hepatitis with jaundice and CMV reactivation. In contrast, EBV reactivation is associated with a milder form of the disease. However, in the latter cases there is a higher rate of development of autoimmune diseases such as type 1 diabetes mellitus [24] and autoimmune hypothyroidism [1]. These autoimmune diseases can occur even several years after resolution of the DIHS. Complications are rare; they include limbic encephalitis, thyroid diseases, renal failure, syndrome of inappropriate secretion of antidiuretic hormone [25], spleen rupture [26], eosinophilic colitis [27], eosinophilic esophagitis [28], and myocarditis [29]. A recent report has described a case of lethal enterocolitis associated with CMV reactivations in the setting of DIHS [30].

### Diagnosis

Although the individual symptoms of DIHS are not specific and can suggest another disease (e.g. lymphoproliferative disease, connectivitis, etc.), their combination associated with HHV-6 infection and the concomitant use of a drug can indicate the diagnosis. A Japanese group has devised a list of criteria for a diagnosis of DIHS [21,31]: 1. Maculopapular rash developing >3 weeks after starting therapy with a limited number of drugs; 2. Prolonged clinical symptoms after discontinuation of the causative drug; 3. Fever (>38°C); 4. Hepatitis (ALT >100 U/L) or renal involvement; 5. Leukocyte abnormalities (a. Leukocytosis (>11 × 10^9^/L), b. Atypical lymphocytosis (>5%), c. Eosinophilia (1.5 × 10^9^/L)); 6. Lymphadenopathy; 7. HHV-6 reactivation. The presence of 7 criteria is indicative of typical DIHS. The presence of 5 criteria is indicative of atypical DIHS [21,31].

Reactivation of HHV-6 or other herpesviruses can be assessed by PCR or specific IgM. However, as herpesvirus infections associated with DIHS represent likely a reactivations from latency, most patients would probably express specific IgM over a relatively short time window (since the secondary T helper cell response kicks in more rapidly, driving a more rapid isotype conversion for newly arisen circulating naïve B lymphocytes). Moreover it is noteworthy that in the course of DIHS a dramatic decrease in immunoglobulin production occurs [31]. Therefore PCR seems to be a more reliable marker for current herpesvirus activity than specific IgM.

### Therapy

DIHS is treated with oral corticosteroids (1-1.5 mg/kg body weight/day). It is noteworthy that rapid tapering of corticosteroids is associated with reactivation of the syndrome [32] as occurred in our patient. Intravenous IgG and plasma exchange have been successful in cases in which the disease did not regress under corticosteroids [33]. Intravenous immune globulin may act by forming immune complexes that block IgG Fc receptors, by neutralizing autoantibodies, and by controlling virus infection.

In some case reports, N-acetylcysteine together with immune globulin was successfully used [32].

## Conclusions

The case reported herein demonstrates that comprehensive medical history-taking and the search for rare causes of fever can lead to complete resolution of a very difficult case. The most relevant issue related to DIHS is its identification. Physicians should suspect it in all cases of fever that appears several weeks after the administration of a drug (usually an antiepileptic drug). Treatment is usually successful once the condition has been diagnosed. A critical issue is the duration of treatment. What this case clearly shows is that steroidal tapering should be very slow. However no study has been carried out to assess the ideal length of the treatment.

We underline that acyclovir, started as empirical treatment due to worsening conditions of the patient, likely had no role in the resolution of the symptoms. HHV-6 DNA was positive in the blood the day after acyclovir withdrawn.

DIHS is a paradigmatic disease in which drugs and infections interact with the immune system to generate a syndrome constituted by a lymphoproliferative disease, an autoimmune disease, an infectious disease and an allergic reaction. The study of the pathogenesis of DIHS could shed light on the pathogenesis of several other immune diseases.

## Competing interests

The authors declare that they have no competing interests.

## Authors' contributions

IG was part of the medical team of the patient and drafted the manuscript. MT was part of the medical team of the patient. GB was the head of the medical team of the patient and revised the manuscript. All authors read and approved the final manuscript.

## Consent

Written informed consent was obtained from the patient for publication of this case report.

## Pre-publication history

The pre-publication history for this paper can be accessed here:

http://www.biomedcentral.com/1471-2334/10/49/prepub
